# Intensity and Inhalation of Smoking in the Aetiology of Laryngeal Cancer

**DOI:** 10.3390/ijerph8040976

**Published:** 2011-04-01

**Authors:** Heribert Ramroth, Andreas Dietz, Heiko Becher

**Affiliations:** 1 Institute of Public Health, University of Heidelberg, Im Neuenheimer Feld 324, D-69120 Heidelberg, Germany; E-Mail: heiko.becher@urz.uni-heidelberg.de (H.B.); 2 Department of Otorhinolaryngology, Head and Neck Surgery, University of Leipzig, Liebigstrasse 10-14, D-04103 Leipzig, Germany; E-Mail: andreas.dietz@medizin.uni-leipzig.de

**Keywords:** laryngeal cancer, smoking, inhalation, smoking intensity, puffing, quitting smoking

## Abstract

The carcinogenic effect of smoking on laryngeal cancer is well established; however, the risk pattern for detailed smoking characteristics is less clear. Thus, the aim of this analysis was to quantify the impact of different inhalation behaviours on the risk of laryngeal cancer. We conducted a population-based case control study in Germany, frequency-matched for sex and age, using a standardized questionnaire covering lifelong smoking details, including age at start, time since quitting, types of smoking products, duration, intensity and inhalation behaviour. We found higher risks for increasing duration and intensity of smoking. A clear dose-response relationship was found in all inhalation subgroups, *i.e.*, not only for deep inhalers, but also for those puffing on a cigarette. Clearly reduced risks could be observed for quitting smoking. Changing inhalation habits might be considered as a first step to reducing the risk of developing laryngeal cancer. However, the best way to effectively reduce laryngeal cancer risk is to quit smoking.

## 1. Introduction

Smoking behaviour is the most important risk factor for a range of cancers, especially for lung, head and neck cancer [1,2]. The carcinogenic effect of smoking on laryngeal cancer is well established; however, the risk pattern for detailed smoking characteristics is less clear. In contrast to cigarette smoking, cigar and pipe smoking are often associated with different smoking behaviour, as it is assumed that cigar and pipe smokers normally do not inhale the smoke [3]. Thus, different types of inhalation are more likely to be defined by type of smoking than by responses to questions about inhalation behaviour [4]. Additionally, it is assumed that women do not inhale as deeply as men do, thus possibly resulting in an additional independent factor lowering risk of developing head and neck cancer [5,6]. Women are more likely to consume “light” cigarettes, though it has been stated that “light” cigarette users puff more frequently and deeply to achieve the same level of nicotine intake [7].

A recent study of the EPIC working group investigated the effect of pipe and cigar smoking and different characteristics of smoking behaviour, such as types of smoking compounds, duration of smoking, smoking intensity and smoking inhalation [8]. Detailed analyses were provided for tobacco related cancer sites such as cancer of the lung, bladder, liver, stomach, pancreas, kidney, colorectal cancer and the overall group of cancer of the upper aero-digestive tract. However, they did not investigate laryngeal cancer. Thus, the aim of this analysis was to quantify the impact of different inhalation behaviours on laryngeal cancer.

## 2. Methods

This population-based case-control study was conducted in Germany between 1998 and 2000 with 257 histological confirmed cases (236 males, 21 females, response rate 89.2%). The study region covered a population of about 2.7 million in South-West Germany, comprising the cities of Heidelberg, Mannheim, Ludwigshafen, Darmstadt and Heilbronn. Cases and controls were restricted to Germans aged up to 80 who were registered as citizens in the study region. Population controls were selected randomly from the population registries of the study area, and were 1:3 frequency-matched for age and sex (response rate 62.4%) [9]. Risk factors were obtained with face-to-face interviews conducted by five interviewers using a standardized questionnaire. Details for assessment and results of tobacco and alcohol consumption have been described elsewhere [10]. In producing the overall description of an individual’s smoking behaviour, each smoking period lasting longer than 6 months with a relatively constant smoking pattern during an individual’s lifetime was considered. Types of smoking were distinguished as cigarette, cigarillo, cigar and pipe smoking from the age at start of smoking up to the date of interview.

**Figure 1.**
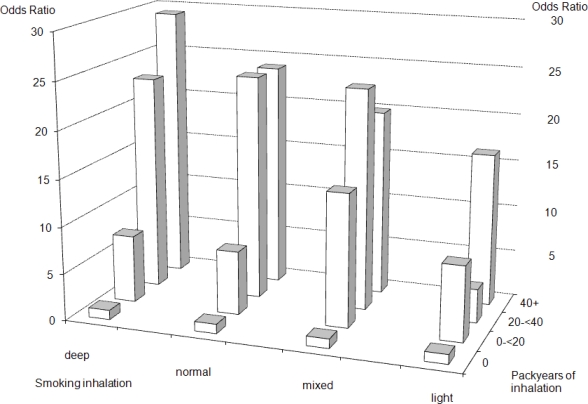
Odds ratios for different types of inhalation (deep, normal, light, mixed) among cigarette-only smokers.

In our analyses, we also took into account information about the depth of smoking inhalation. The questionnaire distinguished between three inhalation categories for each smoking period, for any given smoking product: deep inhalers (“tief inhaliert”), normal smokers (“flach inhaliert”) and puffers (“nicht inhaliert, nur gepafft”)—the latter group will be referred to also as “light” inhalation. To provide comparable results to similar papers on smoking and cancer, in our models we present results for being smoker (yes/no), years of smoking, and type of inhalation (light/puffing, middle, deep, mixed). We present two models on level of smoking inhalation in terms of packyears (py), where one packyear is identical to 20 cigarettes smoked daily for one year. In one model the level of inhalation (py) is categorised into the four levels: 0 py, 0 < … < 20 py, 20 <= … < 40 py, more than 40 py (Figure 1). In a second model packyears are included as a continuous variable, devided by 10 to provide readable results (Table 3). Daily alcohol consumption was calculated from the alcohol data obtained by interview (daily, weekly and monthly alcohol consumption 10 years before interview for all comon alcoholic beverages), assuming the follwing ethanol content: beer 5%, wine, fruit wine or sparkling wine 10%, aperitif and liquors 20%, and sprits 40%. A drink was calculated as containing 20 mL ethanol, equivalent to 1 big bottle of German beer or 1 quarter litre of wine. Only a small percentage (<1%) of smoking and alcohol information was missing. Missing values were replaced by the mean value of the study participants from the same age and sex category.

All odds ratios (OR) given are based on a logistic regression model conditioned on a sex × age classification (five-year age groups) [11]. For the analyses we followed different adjustment strategies to present odds ratios with 95% confidence intervals (95%-CI): In the first adjustment the variable “quitting smoking” (binary: “within the last 2 years before interview” *versus* “quitting later or still smoking”) was modelled together with the smoking or inhalation variable (OR_1_ and 95%-CI_1_). A second adjustment, indicated as OR_2_ and 95%-CI_2,_ additionally included alcohol consumption (unit: 2 drinks per day) and education (in years of education). The interpretation of the risks is based on values adjusted for all three variables, *i.e.*, OR_2_ and CI_2_. P-values below 0.05 were considered statistically significant.

Chi-square tests were used to describe differences between sex, time since quitting smoking, type of inhalation and education. T-Tests were used to describe differences between the continuous variables age, packyears and alcohol consumption. Trend tests were performed using the categorical variables as distinct in the model. All analyses were performed using SAS statistical software (Version 9.2).

## 3. Results

Table 1 describes the distribution for the socio-demographic and lifestyle variables separately for cases and controls.

Only 5.1% of the cases were lifelong non-smokers, in contrast to nearly one third of the controls, with a smoking duration of more than 40 years for more than half of the cases and only 17.6% of the controls. In both groups, cigarette smoking was the dominant type of smoking—95.5% of the smoking cases and 85.4% of the smoking controls were cigarette only smokers—with no cigar, cigarillos or pipe exclusive smokers among cases and only 3% of controls. Thus, no detailed analyses for different types of tobacco products can be presented here. However, information on inhalation behaviour could be obtained directly from questions pertaining to the different types of inhalation. Here, two-thirds of cases reported having been deep inhalers in contrast to 41.6% of controls. Differences between the two groups were also observed in terms of daily alcohol consumption and years of education (see Table 1).

To ease comparison with other papers, we present models for smoking (yes/no), smoking duration and inhalation, with deep inhalers serving as the reference category for degree of inhalation (Table 2). Here, light inhalation showed a significantly decreased risk compared to deep inhalers (OR = 0.22, 95%-CI: 0.09–0.55). In the group of exclusive cigarette smokers, this result was only borderline significant (p = 0.05), likely due to small numbers (data not shown).

Table 3 and Figure 1 present results for more detailed levels of inhalation. Here, a clear trend for increasing odds ratios can be seen for increasing levels of inhalation, present also in the lowest category of light inhalers, though the light inhalation category consisted only of small numbers (Figure 1). Only slight differences were observed between cigarette-only smokers and all smokers, thus, Figure 1 shows only the odds ratios for cigarette-only smokers.

The best model fit (likelihood ratio test) was obtained coding all inhalation levels as continuous variables in terms of packyear (Table 3). The effect of the continuous variables was observed in each of the different inhalation levels. However, significant ORs for cigarette smokers could only be seen adjusting for quitting smoking in the group of light smokers (OR_1_), possibly due to the small numbers. This model also presents the ORs for time since quitting smoking (OR = 0.43; 95%-CI: 0.29–0.62), alcohol consumption (OR = 1.3 per 2 drinks per day; 95%-CI: 1.2–1.5) and education, although all other models were also adjusted for those variables.

## 4. Discussion

Our study confirms a carcinogenic effect not only for the pattern of normal smoking or deep inhalation but also for merely puffing on smoking products, though showing reduced risks for light inhalation in comparison to deep inhalers. As puffing on cigarettes is still regarded as less dangerous, the aim of the present study was to analyse the data under this aspect, especially regarding the still increasing trend of incidence rates of laryngeal cancer among women in the German population [12,13]. As this is one of the biggest European studies on laryngeal cancer, we were able to provide detailed results for different inhalation behaviours of smoking. We found higher risks for increasing duration and intensity of smoking as well as a clear dose-response relationship in all inhalation sub groups. Effects were observed not only for deep inhalers, but also for those puffing on a cigarette, though the latter group was based on small numbers only. Clearly reduced risks could be observed among those who quit smoking, confirming our previous results [10,14]. Thus, changing inhalation habits might theoretically be considered a first step in reducing the risk of developing laryngeal cancer; however it is very unlikely that a smoker would switch to puffing after years of deep inhalation. Considering different adjustment strategies, we followed the most used method to adjust for alcohol consumption and education only [8]. However, a better model fit could be observed using quitting smoking as an additional adjusting variable. Looking at the results without adjustment for quitting smoking, the effect in the presented categories for light inhalation was sometimes only borderline statistically significant. However, additional adjustment for quitting smoking might be an important strategy here, as light smokers are probably those with the highest potential to quit smoking. In further analyses we separated the packyear variable and the inhalation variable, thus analysing the inhalation effect per se. Although not statistically significant for any of the inhalation levels, we could see the same pattern.

Unfortunately, we could not analyse the effect of different types of smoking, due to the small number of those reporting exclusive pipe, cigar and cigarillo smoking. Thus the analysis presents mainly the pattern in a population of exclusive cigarette smokers, although Germany is considered to be among the countries with a high market for cigars [8]. Additionally, we could not verify differences in gender patterns, due to the low number of women (8–9%) in our study. Interestingly, the light smoker group was not dominated by women.

Misclassification of inhalation might possibly change our results, especially, as some results are based on small numbers only. Thus non-differential missclassification cannot be ruled out since the self-reported exposures took place several decades ago, and we did not measure the validity of self-reported inhalation. However, previous studies suggested that self-reported inhalation correlates well with carboxyhaemoglobin saturation levels [8,15].

Though this study shows that light-inhalation smoking is lower-risk than deeper inhalation, it still confirms that quitting smoking is best. As we observed increasing ORs with increasing amounts of smoking in all inhalation groups and a reduction to less than half the risk of laryngeal cancer for those who quit smoking as compared with current smokers, quitting smoking seems still to be the best strategy to reduce laryngeal cancer risk.

## Figures and Tables

**Table 1.** Distribution of variables.

**Variable**	**Category**	**Cases N (%)**	**Controls N (%)**	**Cases *vs.* Controls Comparison (p-value)**
Sex	Males	236 (91.8)	702 (91.3)	
	Females	21 (8.2)	67 (8.7)	0.7884^1^

Age (years)	... < 50	20 ( 7.8)	64 ( 8.3)	
	50 <= … < 60	76 (29.6)	227 (29.5)	
	60 <= … < 70	94 (36.6)	253 (32.9)	
	70 <= …	67 (26.1)	225 (29.3)	0.7652^2^

Type of Smoking	Never	13 ( 5.1)	233 (30.3)	
	Cigarettes only smoker	234 (91.0)	458 (59.5)	
	Cigars only smoker	0 ( 0.0)	9 ( 1.2)	
	Cigarillos only smoker	0 ( 0.0)	5 ( 0.7)	
	Pipe only smoker	0 ( 0.0)	10 ( 1.3)	
	mixed	10 ( 3.9)	54 ( 7.0)	<0.0001^1^

Inhalation	Deep inhalers	170 (66.1)	320 (41.6)	
	Normal smoking	40 (15.6)	107 (13.9)	
	Light inhalation	7 ( 2.7)	51 ( 6.6)	
	Mixed inhalation	27 (10.5)	58 ( 7.5)	<0.0001^1^

Deep inhalers (packyears)	0	87 (33.9)	449 (58.4)	
	0 < … < 20	18 ( 7.0)	144 (18.7)	
	20 <= … < 40	60 (23.3)	93 (12.1)	
	40 <= …	92 (35.8)	83 (10.8)	<0.0001^2^

Normal smoking (packyears)	0	217 (84.4)	662 (86.1)	
	0 < … < 20	8 ( 3.1)	68 ( 8.8)	
	20 <= … < 40	14 ( 5.4)	21 ( 2.7)	
	40 <= …	18 ( 7.0)	18 ( 2.3)	<0.0001^2^

Light inhalation (packyears)	0	250 (97.3)	718 (93.4)	
	0 < … < 20	3 ( 1.2)	40 ( 5.2)	
	20 <= … < 40	1 ( 0.4)	9 ( 1.2)	
	40 <= …	3 ( 1.2)	2 ( 0.3)	0.003^2^

Mixed inhalation (packyears)	0	230 (89.5)	711 (92.5)	
	0 < … < 20	3 ( 1.2)	16 ( 2.1)	
	20 <= … < 40	12 ( 4.7)	24 ( 3.1)	
	40 <= …	12 ( 4.7)	18 ( 2.3)	0.1166^2^

Smoking duration (years)	0	13 ( 5.1)	233 (30.3)	
	0 < … < 20	14 ( 5.4)	152 (19.8)	
	20 <= … < 40	99 (38.5)	249 (32.4)	
	40 <= …	131 (51.0)	135 (17.6)	<0.0001^2^

Quitting smoking (years)	Within the last 2 years	176 (68.5)	383 (49.8)	
	More than 2 years ago	81 (31.5)	386 (50.2)	<0.0001^1^

Alcohol consumption (ml Ethanol)	0	18 ( 7.1)	39 ( 5.1)	
	0 < … < 25	49 (19.2)	303 (39.5)	
	25 <= … < 50	47 (18.4)	178 (23.2)	
	50 <= ... < 75	43 (16.9)	127 (16.5)	
	75 <= …	98 (38.4)	121 (15.8)	<0.0001^2^

Years of Education (years)	<9	224 (87.2)	479 (62.3)	
	9	18 ( 7.0)	120 (15.6)	
	10+	15 ( 5.8)	170 (22.1)	<0.0001^2^

1 Chi-square test;

2 T-Test for continuous variable.

**Table 2.** Distribution and odds ratios for categorical smoking and inhalation variables.

**Variable**	**Category**	**Cases N (%)**	**Controls N (%)**	**OR_1_**	**CI_1_**	**OR_2_**	**CI_2_**
Smoker (yes/no)	Non-Smoker	13 (5.1)	233 (30.3)	1	-	1	-
	Smoker	244 (94.9)	536 (69.7)	17.1	(9.2, 31.5)	17.1	(8.8, 33.4)

Smoking duration * (years)	0	13 (5.1)	233 (30.3)	1	-	1	-
	0 < ... < 20	14 (5.4)	152 (19.8)	3.7	(1.5, 8.8)	4.5	(1.8, 11.4)
	20 <= ... < 40	99 (38.5)	249 (32.4)	11.7	(6.0, 22.7)	12.7	(6.2, 25.8)
	40 <= ...	131 (51.0)	135 (17.6)	22.7	(12.0, 42.8)	21.6	(10.9, 43.0)

Inhalation *	deep	170 (66.1)	320 (41.6)	1	-	1	-
	middle/mixed	67 (26.1)	165 (21.5)	0.67	(0.47, 0.97)	0.73	(0.50, 1.1)
	light	7 (2.7)	51 (6.6)	0.23	(0.10, 0.53)	0.22	(0.09, 0.55)
	Non-smoker	13 (5.1)	233 (30.3)	0.05	(0.02, 0.09)	0.05	(0.02, 0.10)

OR: Odds Ratio; 95%-CI: 95%-confidence interval; OR_1_, 95%-CI_1_: adjusted for quitting smoking (yes/no); OR_2_, 95%-CI_2_: additionally adjusted for alcohol consumption (drinks per day) and education (years);

* p-values for trend, both for OR_1_ and OR_2_ < 0.0001^2^.

**Table 3.** Distribution and odds ratios for continuous inhalation variables (cigarette smoking only).

**Variable**	**Category**	**Cases N (%)**	**Controls N (%)**	**OR_1_**	**CI_1_**	**OR_2_**	**CI_2_**
Non-Smokers		13 (5.1)	233 (30.3)	1	-	1	-

Inhalation (per 10 packyears)	light	51 (6.6)	7 (2.7)	1.3	(1.0, 1.8)	1.2	(0.85, 1.6)
	normal	107 (13.9)	40 (15.6)	1.5	(1.3, 1.7)	1.4	(1.2, 1.6)
	deep	320 (41.6)	170 (66.1)	1.5	(1.4, 1.6)	1.4	(1.3, 1.5)
	mixed	58 (7.5)	27 (10.5)	1.4	(1.2, 1.6)	1.3	(1.1, 1.5)

Quitting smoking	<2 years	383 (49.8)	176 (68.5)	1	-	1	-
	2+ years	386 (50.2)	81 (31.5)	0.43	(0.30, 0.61)	0.43	(0.29, 0.62)

Alcohol consumption	2 drinks per day	-	-	-	-	1.3	(1.2, 1.5)

Years of education	<9 years	224 (87.2)	479 (62.3)	-	-	1	-
	9 years	18 (7.0)	120 (15.6)	-	-	0.48	(0.27, 0.85)
	10+ years	15 (5.8)	170 (22.1)	-	-	0.30	(0.16, 0.56)

OR: Odds Ratio; 95%-CI: 95%-confidence interval; OR_1_, 95%-CI_1_: adjusted for quitting smoking (yes/no); OR_2_, 95%-CI_2_: additionally adjusted for alcohol consumption (drinks per day) and education (years).
